# Characterising the Human Milk Microbiota of Indian Mothers: Prospects for Probiotic Discoveries and Antimicrobial Peptides

**DOI:** 10.1155/ijpe/4819511

**Published:** 2025-04-17

**Authors:** H. Anil, Somashekhar M. Nimbalkar, Chaitanya Joshi, Anju Kunjadiya, Axil Patel, Reshma Pujara, Krutarth Raval, Satyamitra Shekh, Priyanka Dalwadi, Dipen V. Patel

**Affiliations:** ^1^Department of Neonatology, Pramukhswami Medical College, Bhaikaka University, Anand, Gujarat, India; ^2^Department of Science and Technology, Gujarat Biotechnology Research Center, Gandhinagar, Gujarat, India; ^3^Postgraduate Department of Applied and Interdisciplinary Sciences (IICISST), Sardar Patel University, Anand, Gujarat, India; ^4^Central Research Services, Bhaikaka University, Anand, Gujarat, India

**Keywords:** antimicrobial peptide, human milk, microbiome, probiotics

## Abstract

**Background:** The human milk microbiome is vital in the formation of the newborn microbiome and affects various health outcomes. Probiotics prevent severe necrotizing enterocolitis in neonates, but uncertainty about their safety is the obstacle to their use. Probiotic organisms and antimicrobial peptides derived from probiotic strains in human milk can offer safer options.

**Aim:** This study is aimed at determining the probiotic properties in the human breastmilk microbiome and their potential antimicrobial activity.

**Methods:** Study Design: We conducted a prospective longitudinal study. Participants: The study included 30 mothers, equally divided among gestational ages of < 32 weeks, 32–36 6/7 weeks, and above 37 weeks at the time of delivery. Milk samples were collected and analyzed at three different time points, that is, colostrum, transition milk (7–9 days), and mature milk (after 14 days).

**Outcome:** The microbiome isolated was tested for probiotic and antimicrobial properties.

**Results:** Three hundred and eighty-one bacterial colonies were isolated, of which 38 different species were identified. Of these, *Gemella haemolysans*, *Micrococcus luteus* and *lylae*, and *Staphylococcus hominis* and *warneri* were selected. Few showed bile salt, phenol, and NaCl tolerance, but none showed tolerance to pH. Antimicrobial activity was not seen when isolates or protein extracts were tested against the pathogen. Enhancement in the zone of clearance was seen when a combination of protein and antimicrobial agents was tested compared to antimicrobial alone. The zone of clearance was seen even at one-tenth of the standard concentration of amphotericin B when combined with protein extract.

**Conclusion:** The non-*Lactobacillus* strains tested showed few probiotic properties. Though the isolates did not exhibit antimicrobial properties, the protein extracted from them enhanced the potency of antimicrobial agents.

## 1. Introduction

In recent times, human milk has been found to be inheriting a spectrum of microbiomes. These microbiome when entering the infant helps in the development of the infant's gut and airway microbiome. It also benefits the immune system (including the development of tolerance) and other health outcomes [1–3].

Human milk has proven benefits, including reduced risk of infections related to the respiratory tract; allergic disorders like atopic dermatitis and asthma; chronic diseases like obesity, Type 2 diabetes, necrotizing enterocolitis (NEC), especially in preterm newborns; sudden infant death syndrome; and diarrheal disorders [4, 5].

The milk microbiome targets the intestinal epithelia, influencing nutrient absorption, permeability of the mucosal membrane, proliferation of epithelial cells, altering the gut microbiota, and regulation of cytokine production. In addition, it has a crucial role in the maturation of the enteric nervous system, mucosal, and gut immune system [6].

Now, the use of probiotics has become integral in preterm care as it prevents severe NEC and all-cause mortality [7]. Probiotic formulations are available in the current market, but uncertainty about their safety forms a major obstacle to their use. Human milk, being natural, can be a promising source of probiotic organisms.

India represents a unique demography with diverse dietary practices, cultural traditions, and regional variations. The milk microbiome of Indian women is likely to reflect this rich diversity of microbial strains. Understanding their probiotic potential can shed light on their health benefits and pave the way for developing targeted interventions.

Lactobacilli species have been known to produce bacteriocins like lactobrevin, lactoacidin, and acidophilin, which, by lowering the gut pH, affect the growth of harmful bacteria and also are potential toxins to microbes by themselves [8, 9]. Lots of studies have been conducted on *Lactobacillus* species, but less is known about other species.

## 2. Aims and Objectives

1. To determine the probiotic properties of the human breastmilk microbiome.
2. To identify their potential antimicrobial activity.

## 3. Material and Methods

A prospective longitudinal study was conducted in a tertiary care center, between August 2022 and April 2023. Mothers who were admitted to the postnatal ward and NICU were enrolled after obtaining consent. Mothers with mastitis and laboratory or clinically diagnosed sepsis, or those extramurally delivered mothers admitted after 72 h of delivery, were excluded.

This study being a pilot study, we included 30 mothers, equally divided among gestational ages of < 32 weeks, 32–36 6/7 weeks, and above 37 weeks at the time of delivery. The milk samples were collected at three different periods of the postpartum day: colostrum (2–3 days), transitional milk (7–9 days), and mature milk (after 2 weeks).

### 3.1. Sampling Procedure

The samples were collected into a sterile container under a sterile technique by the mothers using sterile gloved hands. The nipple and areola region were cleaned with soap and water. The staff nurse trained in routine care of mothers assisted them. The samples were then immediately sent for culture within 2 h, and the temperature was maintained between 2°C and 8°C using ice packs. Further processing occurred the same day.

The pour plate technique was used to isolate the organisms. One-milliliter aliquots of the samples were plated into nutrient agar (NA), BHI (brain heart infusion) agar, and MRS (Man, Rogosa, and Sharpe) agar media (pH 6.2). The plates were incubated at 37°C for 2–3 days under aerobic and anaerobic conditions (in anaerobe jar using Oxoid AnaeroGen compact) in order to cultivate aerobes, strict anaerobes, and facultative anaerobes [10]. The detailed process of the above technique has been mentioned in File S1 (Online Resource 1). Different bacterial species were identified by MALDI-TOF MS and were cross-verified by 16s RNA sequencing technique.

Then, potential probiotic organisms were subjected to basic probiotic screening tests. Isolates were inoculated in 5 mL of BHI broth. It was modified with bile salt (0.7%), NaCl (2% and 4%), pH (2, 3, and 4) adjusted with 0.1 M HCl, and phenol (0.2%, 0.3%, and 0.4% phenol). Samples (0.1 mL) were collected from these tubes after 24 h and were plated on BHI agar and incubated at 37°C for 48 h for the determination of viability [11].

These isolates were evaluated for organic acid production by inoculation in BHI broth and incubation at 37°C for 24 h [12]. Their mucin degradation properties were also assessed. NA and Sabouraud agar plates, with added 0.3% mucin, were prepared, and spots of pure isolates were inoculated using a nichrome wire loop [13]. The detailed process of the above technique has been given in File S1 (Online Resource 1).

Further, these isolates were subjected to the following tests:

1. Antibiotic susceptibility test: On the Mueller–Hinton agar plates, 1 mL of freshly prepared culture inoculum (O.D., 0.6–0.8) was spread. Icosa G-I plus antibiotic strip was placed in the middle of the plate and incubated at 37°C for 24 h, and the zones were measured to determine the antibiotic susceptibility [14].
2. Antibacterial screening test: Using spot overlay and stroke overlay techniques, the isolates were tested against pathogens like *Escherichia coli* and *Staphylococcus aureus* and observed for the zone of clearance [14]. The procedure is explained in detail in File S1 (Online Resource 1).
3. Anti-*Candida* activity test using different media: The plates of respective media (Sabouraud dextrose agar (SDA), NA, and BHI agar) were prepared, and the pathogen *Candida albicans* (O.D., 0.6–0.8) was spread on the prepared plates and incubated at 37°C for 3 h. A loop full of freshly prepared pure culture was streaked in a 2 cm line on the prepared plate and incubated at 37°C for 24 h and observed for the zone of clearance [14].
4. Isolation of protein using 80% ammonium sulfate precipitation method: Protein was extracted from the isolates, and the amount of protein concentration was estimated using the Bradford assay (Table 1) and was used in further experiments [15]. Details of the above method have been mentioned in File S1 (Online Resource 1).
5. Antimicrobial screening test of protein using well diffusion method: Culture plates of respective media were prepared according to the selected pathogen (*E. coli* and *S. aureus*—NA plates; *C. albicans*—SDA plate). Pathogens were spread on respective plates, and 6 mm wells were prepared using a cup borer, and the bottom of the well was sealed using 0.1 mL of agar. Protein samples were loaded into wells. Plates were incubated at 37°C for 24 h and observed for the zone of inhibition. [16].
6. Protein samples: Combinational activity: Antimicrobial screening test using well diffusion method: Prepare the plates of respective media and the pathogen as above and add the equal concentration of protein and chloramphenicol/amphotericin B (positive control) in the wells.

The outcome of the study was to determine the probiotic properties of various microbiomes other than *Lactobacillus* species in human breast milk and to know the antimicrobial properties.

## 4. Results

A total of 381 bacterial colonies were isolated, of which 38 different bacterial species were identified by MALDI-TOF MS. These were cross-verified by 16s RNA sequencing technique, and both the results were in agreement with each other.

Of these isolated bacterial species, a literature search was done to know the possible beneficial bacteria. Apart from *Lactobacillus* organisms like *Lactobacillus rhamnosus* and *Lactobacillus gasseri*, the following organisms were noted: *Bacillus cereus*, *Bacillus pumilus*, *Bacillus zhangzhouensis*, *Bacillus altitudinis*, *Enterococcus faecium*, *Gemella haemolysans*, *Micrococcus luteus*, *Micrococcus lylae*, *Staphylococcus hominis*, *Staphylococcus warneri*, *and Weissella cibaria.*

*G. haemolysans* has been shown to inhibit the periodontal pathogen *Porphyromonas gingivalis* [17]. *M. luteus* was noted to improve skin health quality [18]. *S. hominis* showed antibacterial activity against *S. aureus* [19]. As studies on the potential use of *B. cereus*, *B. pumilus*, *B. zhangzhouensis*, and *E. faecium* are available, we subjected the remaining organisms to further tests.

As a part of the probiotic potential assessment, these were tested for tolerance to pH, bile, NaCl, and phenol. The results are depicted in Table 2. They were also tested for organic acid production and found that none of the organisms secreted organic acid and were also tested for mucin degradation, where all the organisms were found to be degrading mucin.

### 4.1. Antibiotic Susceptibility

In the present study, based on the wide range of the antibiotic resistance experiment, the following susceptibility pattern was identified (Table 3).

### 4.2. Antimicrobial Activity of Isolates Against Test Pathogens

Antimicrobial activity was carried out against three pathogens: gram-negative *E. coli*, gram-positive *S. aureus*, and *C. albicans*. None showed antimicrobial activity against the test pathogens.

### 4.3. Antimicrobial Activity of Protein Against Test Pathogens

Antimicrobial activity was conducted against the gram-negative *E. coli*, the gram-positive *S. aureus*, and the yeast *C. albicans*. None of them showed antimicrobial activity.

### 4.4. Combinational Activity of Protein and Antibiotic in Equal Concentration: Antibacterial Screening Test Using Well Diffusion Method

**Figure 1:**
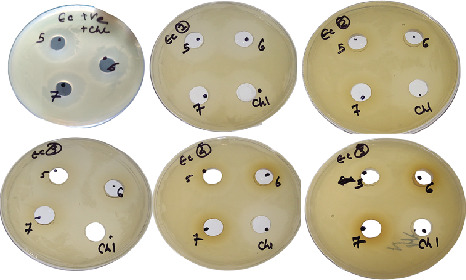
Antimicrobial activity of protein extracts and antibiotic combinations against *E. coli* pathogen at various pH. Note: The first plate is the control, and the other plates labeled are as follows: 1, *Gemella haemolysans*; 2, *Micrococcus luteus*; 3, *Micrococcus lylae*; 4, *Staphylococcus hominis*; 5, *Staphylococcus warneri*. The zone of clearance can be seen in all plates.

**Figure 2:**
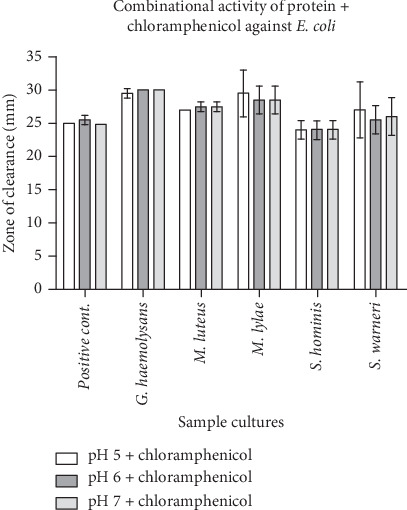
Graphical representation of the combinational activity of protein with antibacterial (chloramphenicol) (equal concentration) and positive control (plain buffer and chloramphenicol equal concentration) against *E. coli*. Note: Positive control contains buffer + chloramphenicol; test sample contains protein isolated in different pH + chloramphenicol. Except for *S. hominis*, all the other organisms showed an increase in the zone of clearance when compared with the control.

**Figure 3:**
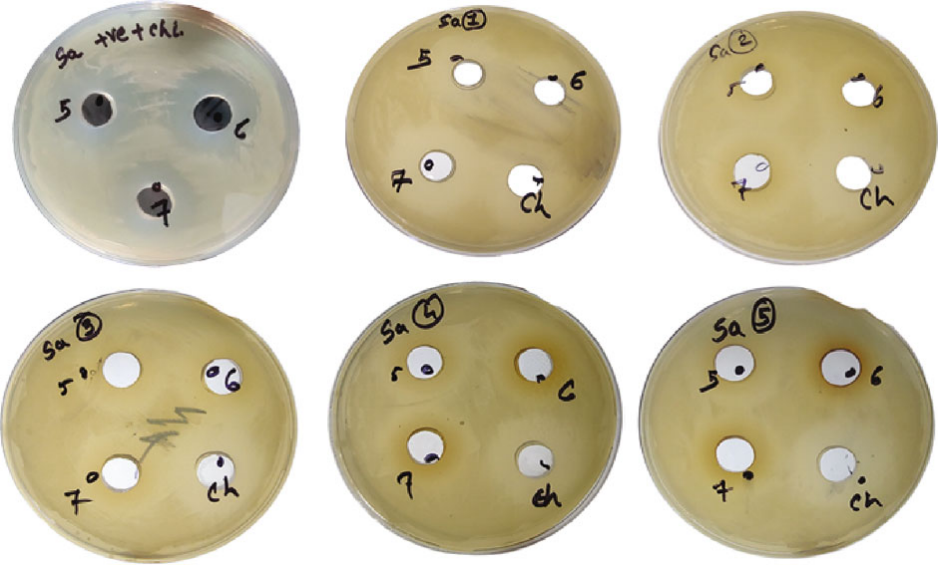
Antimicrobial activity of protein extracts and antibiotic combinations against *Staphylococcus aureus* pathogen at various pH. Note: The first plate is the control, and the other plates labeled are as follows: 1, *Gemella haemolysans*; 2, *Micrococcus luteus*; 3, *Micrococcus lylae*; 4, *Staphylococcus hominis*; 5, *Staphylococcus warneri.* The zone of clearance can be seen in all plates.

**Figure 4:**
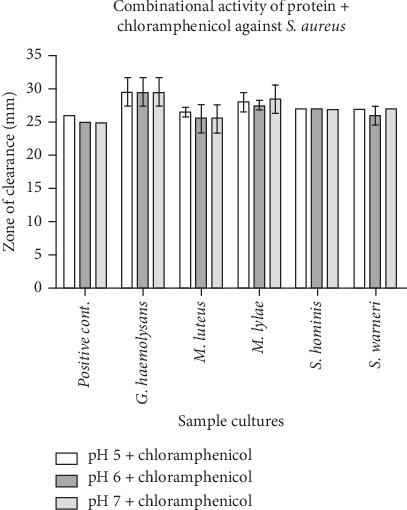
Graphical representation of the combinational activity of protein with antibacterial (chloramphenicol) (equal concentration) and positive control (plain buffer and chloramphenicol equal concentration) against *S. aureus*. Note: Positive control contains buffer + chloramphenicol; test sample contains protein isolated in different pH + chloramphenicol. All the organisms showed an increase in the zone of clearance when compared with the control.

A suitably isolated protein and an equivalent amount of chloramphenicol were combined. The zone of inhibition was seen to be wider in most of the test samples against *E. coli* as compared to the positive control, and the declining zone effect was found to be present in the *S. hominis* sample (Figures 1 and 2). All had a broader zone of inhibition when tested against the *S. aureus* pathogen (Figures 3 and 4).

### 4.5. Combination of Protein and Antifungal: Anti-*Candida* Screening Test Using Well Diffusion Method

**Figure 5:**
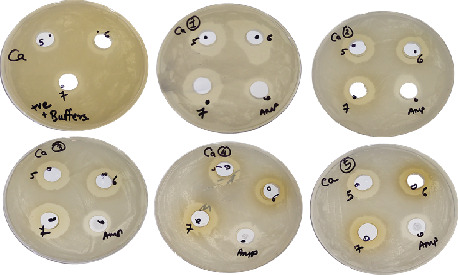
Antimicrobial activity of protein extracts and antifungal combination against *Candida albicans* at various pH. Note: Combinational activity of protein with antifungal (amphotericin B) (equal concentration). The first plate is the control, and the other plates labeled are as follows: 1, *Gemella haemolysans*; 2, *Micrococcus luteus*; 3, *Micrococcus lylae*; 4, *Staphylococcus hominis*; 5, *Staphylococcus warneri*. The zone of clearance can be seen in all plates.

**Figure 6:**
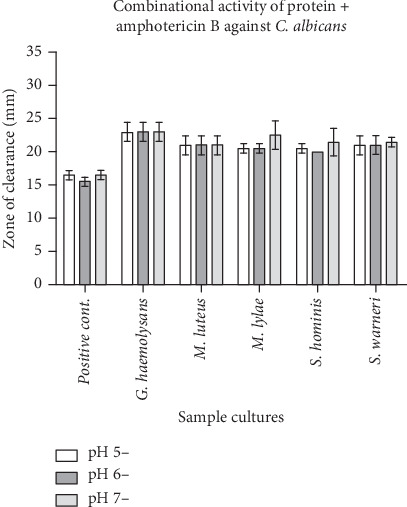
Graphical representation of the combinational activity of protein with antifungal (amphotericin B) (equal concentration) and positive control (plain buffer and amphotericin B equal concentration) against *C. albicans*. Note: Positive control contains buffer + amphotericin B; test sample contains protein isolated in different pH + amphotericin B. All the organisms showed an increase in the zone of clearance when compared with the control.

Promising antifungal activity against the pathogen *C. albicans* was discovered when a protein and amphotericin B were combined. Amphotericin B and the appropriate pH buffer were combined to create the sample preparation for the positive control. The zone of inhibition found in the positive control sample is 15.5 ± 1.5 mm at pH 5, 15 ± 0.5 mm at pH 6, and 16 ± 0 mm at pH 7. A mixture of isolated protein samples with an equal concentration of amphotericin B was prepared. Compared to the positive control, the zone of inhibition was wider in all test samples. *G. haemolysans* had the greatest zone out of all the species, followed by *S. warneri*, *M. luteus*, *M. lylae*, and *S. hominis* (Figures 5 and 6).

To assess the threshold of the combinational antifungal activity of the protein and amphotericin B, a serial decrease in concentration experiment was performed. Three decreasing concentration systems were prepared to assess the antifungal activity with the minimum dosage of amphotericin B. The first system is prepared of protein samples with amphotericin B of equal concentration (1.0), the second system is prepared of protein samples with amphotericin B of half of the concentration (0.5), and the third system is prepared of protein samples with amphotericin B of one-tenth of the concentration (0.1). The volume makeup with respect to the concentration and sample loading in wells is presented in Table 4. The positive control contains 19 *μ*g of amphotericin B + buffer. The zone of clearance against *Candida* using only amphotericin B at various concentrations is shown in Table 5.

### 4.6. Combinational Activity of Protein and Antifungal: Anti-*Candida* Screening Test Using Well Diffusion Method (Amphotericin B—1.0 (Equal Conc.), 0.5 (Half Conc.), and 0.1 (One-Tenth Conc.)]

It was discovered that the test samples could produce a zone that was larger than the positive control in the first system (1.0). Comparing the rest of the sample to the first system, decreasing zones were visible. When the concentration of amphotericin B was reduced to a one-tenth concentration, we had promising outcomes. Here, zones of clearance were noted even at pH 5 and pH 7 with most of the organisms, which were not found when only antifungal was used (Table 6).

## 5. Discussion

Human milk microbiota have recently been receiving attention. Researchers are driven to investigate additional putative probiotic bacteria in human milk as more studies on the potential benefits of *Lactobacillus* strains on newborn health emerge. India, with more than a sixth of the world's population, offers a unique opportunity for research in this area. In our study, *G. haemolysans*, *M. luteus*, *M. lylae*, *S. hominis*, and *S. warneri* species were studied.

Stress tolerance being a major criterion for probiotic selection, the isolates were tested for tolerance against pH, bile, phenol, and NaCl. To reach the small intestine, they have to pass through stressful conditions of the stomach, whose pH can be as low as 1.0 [20, 21]. Here, we used pH of 2, 3, and 4 and tested for tolerance up to 3 h, which is the average gastric emptying time. However, none of the strains showed tolerance; this may be due to lab conditions not accurately mimicking the human body condition or the isolates might have been subjected to other external stressors that could affect the overall viability.

Although the concentration of human bile varies, the mean concentration is believed to be 0.3% *w*/*v*, and the staying time is suggested to be 4 h [22]. Both *Staphylococcus* species showed tolerance to it. All the strains showed tolerance to NaCl; however, only *Staphylococcus* species, *G. haemolysans* and *M. luteus*, showed some tolerance to phenol. This property of phenol tolerance is important for isolates to survive the gastrointestinal conditions, where the gut bacteria have the ability to deaminate aromatic amino acids that are derived from dietary proteins and may lead to the formation of phenols [23]

The strains were further evaluated for their ability to produce organic acid, which could inhibit the growth of pathogenic bacteria, improve gut barrier function, and reduce inflammation [24–26]. However, none showed acid production.

The mucin degradation property of an organism can be both beneficial and harmful. Probiotic bacteria can degrade mucin as it is a source of carbon, nitrogen, and other nutrients. In one of the studies, a mucin-degrading organism is shown to improve gut epithelial barrier function and also has immune modulation property reducing the incidence of inflammatory bowel disease [27]. On the contrary, in one of the studies, *Bifidobacterium* species were able to protect against *E. coli* infection by increasing mucus production in the gut [24], and other studies have shown decreased mucin gene expression leading to adhesion of pathogenic bacteria and increased inflammation [28, 29]. In our study, all tested strains showed mucin degradation.

In antibiotic susceptibility, it was found that some amount of susceptibility was shown to chloramphenicol, whereas the strains showed resistance to most antibiotics (Table 3). This becomes important as the probiotics can transfer the antibiotic resistance gene to other harmful gut bacteria. At the same time, it can offer benefits when simultaneously used with antimicrobials without being destroyed.

The strains were evaluated for antimicrobial activity against *E. coli*, *S. aureus*, and *C. albicans*, but none of them showed antimicrobial activity, nor did the protein extracted from them. *Lactobacillus* species isolated from human milk have shown such properties and are proven worthy probiotic strains [30]. However, the combinational activity of the protein extracted and antibiotic/antifungal showed a response. One of the studies done by Sharma et al. has shown the presence of zone enhancement when probiotic strains were combined with antibiotic agents [31]. This was similar to the findings noted in our study. Another study conducted by Saud et al. showed the antimicrobial activity of probiotic strains present in dairy products against multidrug-resistant bacteria [32].

These findings point towards the possible therapeutic utility of probiotics. In our study, the zone of inhibition was wider with the combinational activity of probiotic peptide and antimicrobial. Another positive finding was a zone of inhibition against *Candida* species at a lower concentration of amphotericin B when combined with peptide, which was not seen with low concentration amphotericin alone.

The emergence of multidrug-resistant bacteria is a major concern because of the indiscriminate use of antibiotics [33]. Antibiotic therapy has also raised concern regarding possible side effects, which demand a safer therapeutic alternative [34]. So, the judicious use of a combination of probiotics and antimicrobials in the treatment of the infection may not only cut the duration of therapy but also reduce the emergence of drug-resistant microorganisms. Antimicrobial activity exhibited by protein extract from probiotic strains has made it a safer option if using probiotic bacteria as a whole is a concern.

## 6. Conclusion

Through this study, we were able to isolate and test various potential probiotic organisms. They were tested for probiotic properties, and all tested organisms showed tolerance to salt, while only *Staphylococcus* species showed bile tolerance. However, none of the test organisms showed pH tolerance. Though the isolates did not exhibit antimicrobial properties, they showed enhancement of antimicrobial activity when a combination of protein and antimicrobial agent was tested and also enhanced antifungal activity of amphotericin B at lower concentrations. These results have compelled us to focus on such properties, which could be a way out from the growing threat of multidrug-resistant microorganisms. With more similar studies and in vivo animal experiments, these findings can be solidified.

## Figures and Tables

**Table 1:** Isolation of protein from organisms using 80% ammonium sulphate precipitation method at various pH.

**No.**	**Protein samples from cultures**		**In *μ*g/100 *μ*L**
1	*Gemella haemolysans*	pH 5	21
		pH 6	19
		pH 7	32

2	*Micrococcus luteus*	pH 5	53
		pH 6	35
		pH 7	73

3	*Micrococcus lylae*	pH 5	72
		pH 6	40
		pH 7	54

4	*Staphylococcus hominis*	pH 5	63
		pH 6	108
		pH 7	119

5	*Staphylococcus warneri*	pH 5	112
		pH 6	143
		pH 7	127

**Table 2:** Tolerance test conducted on isolates at different pH levels and in different concentrations of salt, bile salt, and phenol.

**Sl. no.**	**Probiotic cultures species**	**pH 2/3/4**	**NaCl 2% and 4%**	**Bile salt 0.7%**	**Phenol 0.2%**	**Phenol 0.3%**	**Phenol 0.4%**
1	*Gemella haemolysans*	—	T	—	T	T	—
2	*Micrococcus luteus*	—	T	—	T	T	—
3	*Micrococcus lylae*	—	T	—	—	—	—
4	*Staphylococcus hominis*	—	T	T	T	T	T
5	*Staphylococcus warneri*	—	T	T	T	—	—
6	*Lactobacillus rhamnosus GG* (reference control)	T	T	T	T	T	T

*Note:* T indicates tolerance and — indicates susceptibility.

**Table 3:** Antibiotic susceptibility pattern of study organisms.

**Antibiotic**	**Organisms**					
	**1**	**2**	**3**	**4**	**5**	**6**
Cephalothin (CEP) 30 *μ*g	R	R	S	R	R	I
Clindamycin (CD) 2 *μ*g	R	R	R	R	R	R
Co-trimoxazole (COT) 25 *μ*g	R	R	I	R	R	R
Erythromycin (E) 15 *μ*g	R	R	R	R	R	R
Gentamicin (GEN) 10 *μ*g	R	I	R	I	R	I
Ofloxacin (OF) 5 *μ*g	R	R	S	R	R	S
Penicillin (P) 10 unit	R	R	R	R	R	R
Vancomycin (VA) 30 *μ*g	R	R	R	R	R	R
Ampicillin (AMP) 10 *μ*g	R	R	R	R	R	R
Chloramphenicol (C) 30 *μ*g	I	I	I	I	I	S
Oxacillin (OX) 1 *μ*g	R	R	R	R	R	R
Linezolid (LZ) 30 *μ*g	R	R	R	R	R	R
Azithromycin (AZM) 15 *μ*g	R	R	I	R	R	R
Amikacin (AK) 30 *μ*g	R	R	I	R	R	R
Clarithromycin (CLR) 15 *μ*g	R	R	R	R	R	R
Teicoplanin (TEI) 10 *μ*g	R	R	R	R	R	R
Methicillin (MET) 5 *μ*g	R	R	R	R	R	R
Amoxyclav (AMC) 30 *μ*g	R	R	R	R	R	R
Novobiocin (NV) 5 *μ*g	R	R	R	R	R	R
Tetracycline (TE) 30 *μ*g	R	R	R	I	R	R

*Note:* 1, *Gemella haemolysans*; 2, *Micrococcus luteus*; 3, *Micrococcus lylae*; 4, *Staphylococcus hominis*; 5, *Staphylococcus warneri*; 6, *Lactobacillus rhamnosus GG* (reference). I, intermediate susceptibility; S, complete susceptibility.

Abbreviation: R, resistant.

**Table 4:** The volume made up of combination of protein sample, buffer, and various concentrations of amphotericin B.

**Sample**	**pH**	**Protein sample (19 *μ*g) vol. in *μ*L**	**Make up vol. with buffer (100 *μ*L)**	**Amp B (1.0) vol. in *μ*L**	**Amp B (0.5) vol. in *μ*L**	**Amp B (0.1) vol. in *μ*L**
1	5	87.88	12.12	19	9.5	1.9
	6	100	0	19	9.5	1.9
	7	57.75	42.25	19	9.5	1.9

2	5	35.51	64.49	19	9.5	1.9
	6	53.5	46.5	19	9.5	1.9
	7	25.93	74.07	19	9.5	1.9

3	5	26.23	74.77	19	9.5	1.9
	6	47.29	52.71	19	9.5	1.9
	7	34.96	62.04	19	9.5	1.9

4	5	29.97	70.03	19	9.5	1.9
	6	17.57	82.43	19	9.5	1.9
	7	15.9	84.1	19	9.5	1.9

5	5	16.81	83.19	19	9.5	1.9
	6	13.26	86.74	19	9.5	1.9
	7	14.91	85.09	19	9.5	1.9

*Note:* Sample: 1, *Gemella haemolysans*; 2, *Micrococcus luteus*; 3, *Micrococcus lylae*; 4, *Staphylococcus hominis*; 5, *Staphylococcus warneri.*

**Table 5:** Zone of clearance against *Candida* using only amphotericin B at various concentrations.

**Positive control (only amphotericin B 19 mcg)**	
pH	Zones in mm (Amp 1)
5	15.5 ± 1.5
6	15 ± 0.5
7	16 ± 0
pH	Zones in mm (Amp 0.5)
5	15 ± 1
6	14.5 ± 1.5
7	15.5 ± 0.5
pH	Zones in mm (Amp 0.1)
5	0
6	14.5 ± 0.5
7	0

*Note:* The zone of inhibition found in the positive control of the first prepared system (1.0) is 15.5 ± 1.5 mm in pH 5, 15 ± 0.5 mm in pH 6, and 16 ± 0 mm in pH 7. For the second system (0.5), the zone of inhibition is 15 ± 1 mm in pH 5, 14.5 ± 1.5 mm in pH 6, and 15.5 ± 0.5 mm in pH 7. The third prepared system (0.1) showed zones of 0 mm in pH 5, 14.5 ± 0.5 mm in pH 6, and 0 mm in pH 7.

**Table 6:** Combinational activity of protein and antifungal: Anti-*Candida* screening test using well diffusion method (amphotericin B—1.0 [equal conc.], 0.5 [half conc.], and 0.1 (one-tenth conc.]).

**Sample**	**pH**	**Amp 1 + protein (zone of clearance in mm)**	**Amp 0.5 + protein (zone of clearance in mm)**	**Amp 0.1 + protein (zone of clearance in mm)**
1	5	23 ± 1	17 ± 1	14 ± 0
	6	23 ± 1	16.5 ± 1.5	14 ± 0
	7	23 ± 1	16 ± 0.5	13.5 ± 0.5

2	5	21 ± 1	16.5 ± 0.5	0
	6	21 ± 0.5	16.5 ± 0.5	14.5 ± 0.5
	7	21 ± 0.5	16 ± 1	14 ± 0

3	5	20.5 ± 0.5	16 ± 2	0
	6	20 ± 1	16.5 ± 0.5	0
	7	22 ± 1	16 ± 1	0

4	5	20 ± 0	16 ± 0	14 ± 0
	6	19.5 ± 0	16.5 ± 0.5	15 ± 0
	7	21 ± 0.5	17 ± 0	17 ± 0

5	5	21.5 ± 0.5	16.5 ± 0	16 ± 0
	6	21 ± 1	16.5 ± 0.5	16 ± 0
	7	22 ± 1	16.5 ± 0.5	16 ± 0

*Note:* Zone of clearance is seen when amphotericin is used in one-tenth concentration along with protein at pH 5 and pH 7, which was not seen when amphotericin was used alone. Sample: 1, *Gemella haemolysans*; 2, *Micrococcus luteus*; 3, *Micrococcus lylae*; 4, *Staphylococcus hominis*; 5, *Staphylococcus warneri.*

## Data Availability

The datasets generated and/or analyzed during the current study are available from the corresponding author upon reasonable request.

## Supporting Information

Supporting Information Additional supporting information can be found online in the Supporting Information section. File S1: This contains in detail the methodology of various microbiological procedures used in this study.
