# Supplementary material for: Characterising the Human Milk Microbiota of Indian Mothers: Prospects for Probiotic Discoveries and Antimicrobial Peptides
Source: Int J Pediatr. 2025 Apr 17;2025:4819511. doi: 10.1155/ijpe/4819511 (PMC12021482; doi:10.1155/ijpe/4819511)
Supplement: Supporting Information — Additional supporting information can be found online in the Supporting Information section. File S1: This contains in detail the methodology of various microbiological procedures used in this study. [file 4819511.f1.pdf]

Online resource 1

Decoding Human milk microbiota of Indian mothers: Prospects for Probiotic Discoveries and Antimicrobial Peptides

Bacterial isolation - Pour plate technique was used to isolate the organisms. 1 ml aliquots of the samples were plated into nutrient agar, BHI (brain heart infusion agar) and MRS (Man, Rogosa and Sharpe) agar media (pH 6.2). The plates were incubated at 37 °C for 2-3 days under anaerobic conditions (in anaerobe jar using Oxoid anaerogen compact). After incubation, individual colonies were selected and transferred into sterile broth mediums. The isolates were purified by selecting colonies with streak plate technique. Once the colonies were isolated, identification of isolates were done by MALDI-TOF MS (Bruker, Germany).

Secretion of organic acid - by inoculating in BHI broth and incubating at 37°C for 24h. BHI agar plates with added 0.017 % (w/v) Bromocresol Purple dye were prepared and a spot of loop full colonies of freshly prepared pure culture were incubated in these plates at 37ºC for 24h and observed for the zone of clearance.

Mucin Degradation Assay - Nutrient agar and Sabouraud Agar plates with added 0.3% mucin were prepared and spots of pure isolates were inoculated using a nicrome wire loop. After 48 h of incubation, the plates were stained with 0.1% Amido black T for 30 minutes and further destained with 1.2M acetic acid and observed for the zone of clearance.

Spot overlay: pure isolates were inoculated in BHI broth and incubated at 37°C for 24 h. The sample was centrifuged at 8000 rpm at 20ºC for 10min and the cell pellet was washed with PBS and resuspended in phosphate buffer. Prepared Nutrient agar was evenly distributed on sample

plates and pellets were placed. 100 µl of pathogen samples of E. Coli and Staph Aureus (O.D. - 0.6-0.8) were poured on the sample plates. Incubated these plates at 37ºC for 24 h and observed for the zone of clearance.

Stroke overlay: Pure isolates were inoculated in BHI broth and incubated at 37°C for 24h. The samples were centrifuged at 8000 rpm - 20ºC for 10min, kept the pellet with 0.5 ml of supernatant. Vortexed it and made it slurry. Streak a loop of colonies of freshly prepared pure culture in a 2 cm line on the prepared BHI plate. Prepared SDB agar was evenly distributed on sample plates and the organism was suspended. 100 µl of pathogen sample of candida albicans (O.D.- 0.6-0.8) was added to the sample plates. Incubated these plates at 37ºC for 24 h and observed the zone of clearance.

Isolation of Protein using 80% ammonium sulfate precipitation method - the pure culture of a respective bacterial species was innoculated in 300ml of BHI broth and incubated it at 37ºC for 48h. Supernatant of the inoculated pure culture was removed and collected by centrifuging it at 9500 rpm for 30 min. PMSF (phenylmethylsulfonyl fluoride) was added. 80% of ammonium sulfate was added gradually with continuous stirring with the help of a magnetic stirrer until it dissolved completely. Samples were stored at 4ºC for overnight. Centrifuged at 9500 rpm at 4ºC for 20min and pellet was collected. Dissolved the pellet with the minimum amount of respective buffer - pH5-Acetate Buffer, pH6-Phosphate Buffer, and pH7-Phosphate Buffer. Centrifuged the dissolved pellet at 9500 rpm at 4ºC for 20 min and collected the supernatant. Estimated for the amount of protein concentration using Bradford assay and used it in further experiments.
